# Impact of Family Planning Health Education on the Knowledge and Attitude among Yasoujian Women

**DOI:** 10.5539/gjhs.v4n2p110

**Published:** 2012-03-01

**Authors:** Fariba Mahamed, Saadat Parhizkar, Alireza Raygan Shirazi

**Affiliations:** Public Health Department, Faculty of Health Yasouj University of Medical Sciences (YUMS), Iran; Public Health Department, Faculty of Health Yasouj University of Medical Sciences (YUMS), Iran Tel: 98-741-222-9224 E-mail: parhizkarsa@gmail.com; Environmental Health Department, Faculty of Health Yasouj University of Medical Sciences (YUMS), Iran

**Keywords:** Attitude, Education, Family planning, Knowledge, Yasouj

## Abstract

The aim of this study was to determine the effect of health education on the knowledge and attitude regarding family planning and contraception’s method among the women who obligatory attended the Premarital Counseling Center in Yasouj city, Iran. An experimental study was carried out and a total of 200 women were selected for the study using convenience sampling method among women who attended in the health centre in order to utilize the necessary premarital actions. Respondents were divided by two experimental and control groups randomly. A pre-evaluation was done on the knowledge and attitude on family planning using a structured questionnaire. After which, the health education for experimental group was done within four educational sessions during 4 consecutive weeks and control group underwent traditional education method.

Post evaluation was utilized for any changes regarding their knowledge and attitude among the respondents immediately after the intervention. Independent and paired t-test was used to evaluate the mean knowledge and attitude scores differences among both groups. Results showed that there was a significant improvement in respondents’ knowledge and attitude after educational program in experimental group (p<0.001), while no significant difference was observed in knowledge and attitude of control group. The finding also indicated that age was significantly associated with the level of respondents’ knowledge. These results deal the effectiveness of the educational method. In conclusion, the educational method is effective in increasing the knowledge and improving the attitude of women regarding family planning in Yasouj compared to current used educational method. Future educational programs need to incorporate the features that have been associated with successful interventions in the past, as well as including their own evaluation procedures.

## 1. Introduction

According to World health Organization (WHO) estimates, one in every five people in the world is an adolescent, (between 10 and 19 years of age). With an estimated 1.2 billion adolescents alive today, the world has the largest adolescent population in history (Blum and Nelson, 2005). Of these, about 85% or four out of five young people live in developing countries. Moreover, more than half of the world’s population is below the age of 25 (UNFPA, 1998; WHO, 1999). Young people constitute a significant proportion of the Iranian population (Mohammadi *et al.*, 2006). According to recent census (2006), there were approximately 17.6 million young people aged 15–24, accounting for 25% of the Iranian population (Iran Statistic Center, 2007; Assad and Roudi-fahimi, 2007).

Despite the fact that contraceptive usage has increased over a period of time, there exists a Knowledge Attitude and Practice -gap regarding contraception (Malek-Afzali, 1998; Ramesh *et al.*, 1996). The reasons for not using any family planning methods are lack of knowledge and education, religious belief and fear of side effects. Family planning has two main objectives; firstly, to have only the desired number of children and secondly, proper spacing of pregnancies (Mao, 1999).

Knowledge and practice of family planning is strongly related to higher level of education (Ramesh *et al.*, 1996). In most of the studies it was found that education is the prime influencing factor and education affects the attitudinal and behavioral patterns of the individuals (Sajid and Malik, 2010; Mao, 1999). A number of Knowledge Attitude and Practice survey has been carried out covering different population groups (Dabral S, Malik, 2004; Gautam and Seth, 2001; Takkar *et al.*, 2005; Rao, 2005). Based on a majority of researches, in most countries of the world, female adolescents do not receive formal reproductive health education on time, since their puberty happens earlier than boys. Lloyd (2009:85) contends that all 13–15 year olds should be acquiring “reading and writing fluency for lifelong learning, critical thinking skills, health and reproductive health knowledge and skills for social and civic participation. Nevertheless, it offers a standard to make informed and voluntary decisions in their lives, including their sexual, marital and reproductive lives. Studies have showed that 63.4% of puberty disorders and complications among females were because of their ignorance (Mohammadi *et al.*, 2006). Considering the above research results and the fact that the number of youths in Iran is increasing, it is necessary to educate youths regarding family planning particularly during puberty and before marriage. This study tried to educate female adolescents who tended to married for the first time regarding family planning and find out how such education affects their knowledge and attitude.

## 2. Methods

### 2.1 Study area and Study Period

The purpose of current study was to determine the effect of health education on the knowledge and attitude regarding family planning and contraception’s method among the women who obligatory attended the Premarital Counseling Center in Yasouj city, Iran. The study was conducted in Yasouj city located in the South-East of Iran, which is 800 km away from Tehran, the capital city of the country. The study was carried out in Shahid Ashrafi Isfahani Health Center. The center is a referral center which covered the only Premarital Counseling Center in the city. Iran has laws in place making premarital counseling and screening for Thalasaemia mandatory for all couples before they are given approval to get married. One innovative program provides mandatory premarital sex education courses for engaged couples form 1994. To obtain a marriage license, each couple must attend a short course on sexual and reproductive health issues, ranging from reproductive anatomy to spousal communication. The study was conducted from November 2010 to February 2011.

### 2.2 Study Design, Study Participants and Sampling

**Figure 1:**
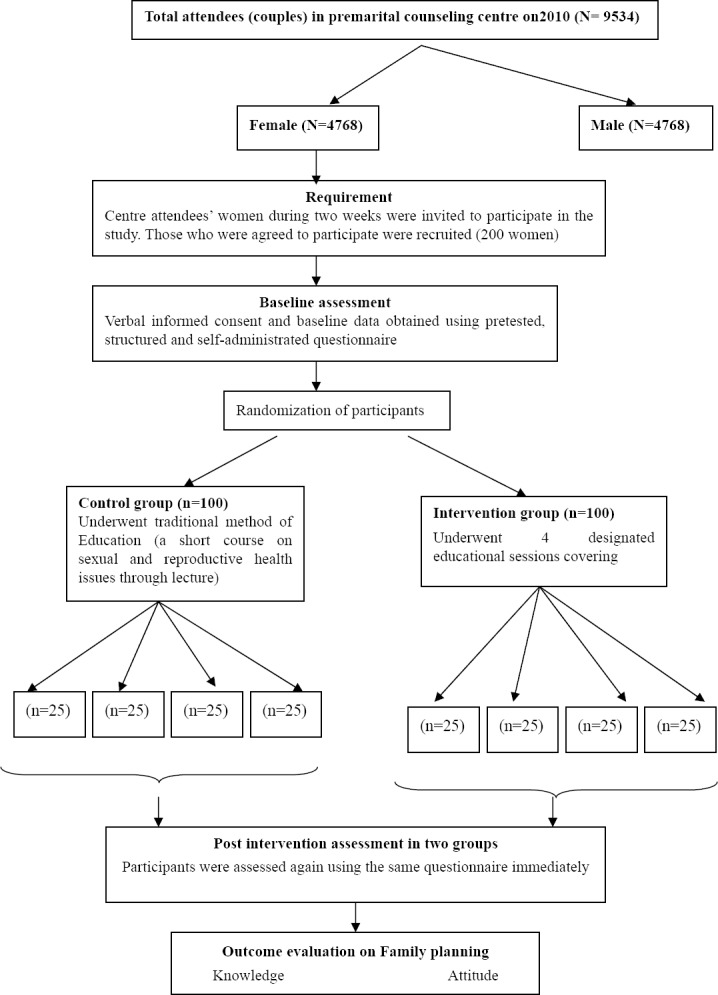
Flow chart on study design and outcome evaluation

An experimental design with an experimental and a control site using pre- and post-intervention measurements was used to investigate the effect of a planned family planning health education on the participants’ knowledge and attitude. The interventional education included in four 1.5 hours sessions covering related contents of contraception methods lectured by six health trainer. To ensure their consistency in educational methods and contents, they were trained seven consecutive days before starting intervention. Furthermore, the trainers also monitored directly using video camera recording which observed by researchers for assuring their consistency in educational methods and contents. The educational method consists of lecture and discussion panel including questions and answers concerning family planning and contraceptive methods which allow the women to express their ambiguity in understanding. Control group underwent traditional educational method which included a single two hours educational session which covering general issues of reproductive health issues consist of family planning, sexual health and spousal communication. Study population including all couples who wish to married and attended to premarital counselling centre during study period. There were 9534 couples who attended to the centre in 2010. A total of 200 women enrolled in the study using convenience sampling method. A convenience sampling method was used to select the study participants, because the application of any of the probability sampling methods would have obstructed the flow of service in the clinic and drastically increased clients’ waiting time. Afterward the selected samples randomly divided by two groups including control and experimental groups according to their registration numbers. The study flowchart illustrated in figure 1.

### 2.3 Research Instruments

Pre and post interventional data was gathered using a structured questionnaire which was developed based on previous studies on Family planning. It consisted of three parts: The first part of the questionnaire was demographic data. The second part of the questionnaire consisted of questions regarding knowledge on family planning and contraception’ methods and the third part comprised of questions regarding attitude toward family planning on both positive and negative statements regarding common issues on family planning with 5 point Likert Scale questions. The knowledge and attitude questions have shown in Table 1 and 2. Content validity was ascertained by an expert panel comprising professionals who were health faculty members, health practitioners and family physicians. Pilot study was carried out on 30 women not included in the study. It was performed as an initial step for the study to check the questionnaire. Some modifications in some questions were done after the pilot study. Its reliability also was determined by alpha-Krunbach (r=0.76) considering the fact that A reliability coefficient (alpha) of. 70 or higher is considered acceptable reliability (Wells and Wollack, 2003).

**Table 1:** Knowledge questions

Definition	Yes	No	Don’t know
Family planning means spacing the birth of children.			
Family planning is the same as abortion.			
Family planning kills babies.			
Family planning is a decision of both husband			
Natural Family Planning method is a way of preventing pregnancy without the use of drugs or devices.			
A condom is a rubber that is inserted into the penis before sexual intercourse.			
Irregular menstruation is one of the possible side effects of IUD.			
A pill is taken by a woman once a week to prevent pregnancy.			
The pills prevent pregnancy by stopping the release of the sperm from the testes.			
Depo Provera injection is given to a woman every month to prevent pregnancy.			
The effect of injection is still present up to 4 months even though injection has been stopped.			
A condom prevents pregnancy by keeping the sperm from getting into the vagina.			
An IUD can travel to the different parts of the body.			
Injection causes abnormal or deformed babies.			
The string of an IUD traps the penis during sexual intercourse			
Pills cause cancer.			
IUD prevents pregnancy by blocking the sperm to come in contact with the egg cell.			
Vasectomy decreases a man’s sexual satisfaction.			
A woman who has undergone tubal ligation cannot do heavy work.			
Tubal ligation involves tying and cutting of both fallopian tubes of a woman.			
A woman who is ligated will stop menstruating.			
Vasectomy is a simple operation that makes a man sterile.			
Vasectomy prevents pregnancy by blocking the sperm from reaching the vagina.			
Vasectomy is a simple operation that makes a man sterile.			

**Table 2:** Attitude questions

Statements	SA	A	NA/ND	D	SD
Do you want to know more about family planning?					
Are you willing to practice family planning?					
Family planning is only for young couples.					
Bringing up a family is a shared responsibility of both husband and wife.					
Family planning improves maternal and child health.					
Family planning is harmful.					
Husbands should also participate in family planning decisions.					
Family planning is important in ensuring a healthy family.					
OCPs can prevent ovarian cysts.					
Depo Provera is suitable for couples at beginning marriage					
Condom can prevent STDs.					
Emergency Contraception is just usable for condom failure.					
Emergency Contraception is usable for unprotected intercourse.					
Weight gain and nausea are OCP’s side effects.					
IUD is suitable for forgetful women.					

SA: Strongly Agree A: Agree NA/ND: Not Agree nor Disagree D: Disagree SD: Strongly Disagree

### 2.4 Study Procedure

After receiving a verbal consent from respondents, pre – interventional assessment was done using the structured questionnaire measuring the respondent’s knowledge and attitude regarding family planning written in Persian. The questionnaire took about 20 minutes to be completed. In order to start study, the experimental group was divided by four equal groups (each group 25 person) who underwent four educational sessions during 4 consecutive weeks. The health education was done by the health trainers which lasted approximately 1.5 hours. Visual aids were used to further guide the respondents during the sessions. Questions were encouraged and a discussion after the lecture followed. Topics discussed during the family planning lecture were the different methods of family planning, its proper use, side effects, advantages and disadvantages. Issues regarding family planning in general were also addressed. These include side effects that one may expect from each method, the fear that some methods can lead to cancer and the complaints regarding the effectiveness of the methods. Some concerns were also regarding the approval of their husbands since some regard family planning as affecting the masculinity of their husbands. Explaining the long –term benefits of family planning on their family’s health and finances was also a goal in the discussions. Furthermore, the traditional education method was done among control group. The knowledge and attitude regarding family planning were assessed immediately after the intervention.

### 2.5 Ethical Considerations

Women were informed of the purpose of the study. It was emphasized that the study was an attempt to improve knowledge and attitude about family planning and contraception methods. Permission to carry out the study was obtained from District Health Center Ethics Committee and Shahid Ashrafi Isfahani Health Center authorities. Questionnaires were assigned unique codes and the results of each individual questionnaire were kept in strict confidence.

### 2.6 Statistical Analysis

Data were entered and analyzed using the Statistical Program for Social Sciences (SPSS) version 16. Each knowledge question was scored 0 for an incorrect response and 1 for a correct response. A summary score was calculated for each subjects’ responses at the pre and Post-intervention data. Each attitude question was scored from 0 to 2. An independent t-test was used to test the differences between the mean scores among groups at the pre-intervention as well as the post-intervention education. Mean scores for experimental group were compared to mean scores for control group. The paired t-test was used to compare the changes in scores from pre- to post-intervention in both groups. The criterion for statistical significance was set at 0.05.

## 3. Results

Demographic and socio-economic information of study adolescents are shown in Table 3. The mean age of subjects was 23.27 (SD=3.96.6). Majority of either experimental or control groups were housewife (97.9 and 93.9 percent respectively) and also most of the respondents’ spouse occupation in both groups worked in private sectors (66 and 60 percent in experimental and control groups respectively). Most of the respondents in experimental group (41.2%) as well as control group (40.4%) have completed high school. Almost of the subjects’ husbands were working in private sector (48.5% and 52.1% in experimental and control group respectively).

**Table 3:** Demographic profile of the respondents

Characteristics	Intervention Group		Control Group	

	No.	%	No.	%
**Age**				
Mean± SD	23.71 ± 4.36		22.84 ± 3.56	
		
**Educational Level**				
Primary School	8	8.3	8	8.5
Secondary School	12	12.5	20	21.3
High school	52	54.1	42	44.7
College/ University	24	25	24	25.5
Total	96	100	94	100
				
**Women Occupation**				
Housewife	93	93.9	92	97.9
Professional	6	6.01	2	2.1
Total	99	100	94	100

There were no significant differences in age, occupation and educational level between two groups. There was a significant (p<0.001) improvement in the level of knowledge regarding family planning among experimental group (enhancement from 3.97±4.04 to 9.67±3.15), while there was no significant difference among control group knowledge. Similarly, the attitude of control group significantly (P<0.001) differed from baseline to end line (4.20±3.20 to 9.73±22.40) which was not significant in control group The results also indicated a significant differences between groups in case of knowledge and attitude after intervention while these level were not different after intervention (Table 4).

**Table 4:** Comparison of groups’ knowledge and attitude’ scores pre and post intervention

	Knowledge			Attitude		
	
	Pre-Test	Post-Test	Within-group comparison	Pre-Test	Post-Test	Within-group comparison
**Intervention Group**	3.97± 4.04	9.67± 3.15	0.001	4.20±3.20	9.73±22.40	0.001
**Control Group**	3.44±3.64	3.35±3.80	NS	3.73±3.23	3.98±3.41	NS
**Between-group Comparison**	NS	0.001	-	NS	0.001	-

Data presented as mean ±SD NS: No Significant

These results deal the effectiveness of the educational method. The finding also indicated that age was significantly associated with the level of respondents’ knowledge, on the other word with increasing the age, the knowledge will increase regarding family planning.

## 4. Discussion

The result demonstrated the poor knowledge and attitude regarding family planning among respondents which was in agreement with the study in the South-East Asia that showed low level of contraception methods information (Dabral and Malik, 2004). Lack of knowledge about contraceptive methods can be a major obstacle in their use (Sajid and Malik, 2010).

The research findings also showed that the mean scores of respondents’ differed significantly before and after intervention among experimental group and mean change of knowledge were about 5.70, while the knowledge score of control group were not significantly different compare to the baseline. It is indicated that family planning education through this method had an impact on improving respondents’ knowledge. On the other word the traditional educational method which used currently is not effective as present method of education. Other studies also proved that health education regarding reproductive health not only increased the respondents’ knowledge, but also improved their healthy behavior (Afghari *et al.*, 2008; Eisenstein *et al.*, 2000; Meuwissen *et al.*, 2006).

The results of the present study revealed that family planning education improved the experimental subjects’ attitude about contraception methods significantly. This may signify that any improvement in a person’s knowledge about something will ensue to a better attitude on it. However, the increase in the mean scores from 4.20 during the pretest to 9.73 during the post proves that family planning education can be a useful tool not just in improving a person’s knowledge but their attitude as well. This is consistent with the results of Wang and his colleagues (2011). Through the health education on family planning, negative attitude on family planning will be changed since promoting a more positive attitude on family planning is a vital step in the actual acceptance of family planning method by people.

The finding also demonstrated that continues education had higher impact on attitude improvement rather than usual educational method which present in a single session of lecture. Young (1996) believes that health education for adolescents strongly affects their attitude toward their abilities and suggests that education should be long term enough to develop their attitude. Poppe (1992) also showed that educational films improve the adolescents’ attitude toward adolescents’ relationships and their knowledge about various safe sexual behaviors.

## 5. Conclusion

Based on this study, health education is an effective way of increasing the Knowledge and Attitude regarding family planning among the women who attended the premarital counseling center in Yasouj. Although current study showed effectiveness of the educational method, but more efforts are needed to change current educational method to be more effective and attractive for couples.
